# Consent for interventions during childbirth: A national population‐based study

**DOI:** 10.1002/ijgo.15830

**Published:** 2024-08-02

**Authors:** Marianne Jacques, Anne Alice Chantry, Anne Evrard, Nathalie Lelong, Camille Le Ray, the ENP2021 Study Group, Camille Le Ray, Nathalie Lelong, Hélène Cinelli, Béatrice Blondel, Nolwenn Regnault, Virginie Demiguel, Elodie Lebreton, Benoit Salanave, Jeanne Fresson, Annick Vilain, Thomas Deroyon, Philippe Raynaud, Sylvie Rey, Khadoudja Chemlal, Nathalie Rabier‐Thoreau, Frédérique Colombet‐Migeon

**Affiliations:** ^1^ Université Paris Cité, Inserm, Center for Research in Epidemiology and StatisticS (CRESS), Obstetrical Perinatal and Pediatric Epidemiology Research Team (EPOPé) Paris France; ^2^ Midwifery University Department Université Paris Cité Paris France; ^3^ Collectif Interassociatif Autour de la Naissance (CIANE) Paris France; ^4^ Maternité Port Royal, Hôpital Cochin Port Royal, Assistance Publique‐Hôpitaux de Paris Université Paris Cité Paris France

**Keywords:** delivery, facility‐based maternity care, informed consent/statistics and numerical data, maternal health services, obstetrics and gynecology, shared decision making, patient‐centred care

## Abstract

**Objective:**

To assess the frequency and determinants of medical interventions during childbirth without women's consent at the population level.

**Methods:**

The nationwide cross‐sectional Enquête Nationale Périnatale 2021 provided a representative sample of women who delivered in metropolitan France with a 2‐month postpartum follow‐up (*n* = 7394). Rates and 95% confidence intervals (CI) of interventions during childbirth (oxytocin administration, episiotomy or emergency cesarean section) without consent were calculated. Associations with maternal, obstetric, and organizational characteristics were assessed using robust variance Poisson regressions, after multiple imputation for missing covariates, and weighted to account for 2‐month attrition.

**Results:**

Women reporting failure to seek consent were 44.7% (CI: 42.6–47.0) for oxytocin administration, 60.2% (CI: 55.4–65.0) for episiotomy, and 36.6% (CI: 33.3–40.0) for emergency cesarean birth. Lack of consent for oxytocin was associated with maternal birth abroad (adjusted prevalence ratio [aPR] 1.20; 95% CI: 1.06–1.36), low education level, and increased cervical dilation at oxytocin initiation, whereas women with a birth plan reported less frequently lack of consent (aPR 0.79; 95% CI: 0.68–0.92). Delivery assisted by an obstetrician was more often associated with lack of consent for episiotomy (aPR 1.46; 95% CI: 1.11–1.94 for spontaneous delivery and aPR 1.39; 95% CI: 1.13–1.72 for instrumental delivery, reference: spontaneous delivery with a midwife). Cesarean for fetal distress was associated with failure to ask for consent for emergency cesarean delivery (aPR 1.58; 95% CI: 1.28–1.96).

**Conclusion:**

Women frequently reported that perinatal professionals failed to seek consent for interventions during childbirth. Reorganization of care, particularly in emergency contexts, training focusing on adequate communication and promotion of birth plans are necessary to improve women's involvement in decision making during childbirth.

## Synopsis

Women often report that professionals do not seek their consent for interventions during childbirth. Care reorganization and specific training are needed to improve birth experience.

## 1 INTRODUCTION

Failure to seek consent to any act of care during childbirth constitutes a failure to meet professional standards of care, according to the commonly accepted classification established by M. Bohren et al.^1^, ^2^ It is outlawed in many countries and is a firmly established element of mistreatment of women in healthcare facilities.^3^ Lack of consent to care during childbirth contributes to negative childbirth experiences.^4^, ^5^ Participation in decision making during childbirth may help protect against perinatal depressive symptoms^6^ and improve the birth experience.^7^ Women strongly expect care that respects their autonomy.^3^, ^8^

Over the past two decades, this issue has been mainly studied in low‐ and middle‐income countries, but the sparse data available in high‐income countries (HICs) suggest that interventions without consent are also common there.^9^ Estimates range from less than 10% for labor induction in an Australian regional survey to 85% for episiotomy in an online survey conducted by a collective of French health‐service user associations in perinatal care.^7^, ^10^, ^11^, ^12^, ^13^, ^14^, ^15^, ^16^, ^17^, ^18^, ^19^, ^20^ The few studies assessing determinants associated with non‐consent interventions inconsistently identified some maternal (e.g., ethnicity and age), obstetric (e.g., nulliparity and mode of delivery), and organizational characteristics.^10^, ^11^, ^13^, ^16^, ^17^ These studies are, however, limited by their designs. None is population‐based on a national scale, with data from both interviews with mothers and medical files (necessary because many women are unaware of the interventions they underwent). Selection bias limits, among other, studies recruiting participants online or through social media.^10^, ^12^, ^13^, ^15^, ^17^, ^18^, ^19^, ^20^ Geographical restriction limits the generalizability of others studies.^7^, ^11^, ^14^, ^16^

More reliable evidence might help improve maternity practices and strengthen respect for women's rights, empowerment, and good treatment during labor and delivery. Accordingly, the 2021 version of the French national perinatal survey (ENP 2021), a nationwide population‐based study with data from medical files and a two‐month follow‐up included, for the first time, questions about consent for interventions during childbirth. Using this data, we aimed to assess the proportion of women who received the most frequent interventions during labor and delivery (oxytocin administration during labor, episiotomy, or emergency cesareans) and reported that their consent was not sought. Then, we examined the associations of failure to seek consent with maternal, obstetric, and organizational factors.

## 2 MATERIALS AND METHODS

### 2.1 Data source

The French National Perinatal Surveys (ENPs) are cross‐sectional studies conducted at regular intervals to assess the health status of mothers and children in France.^21^, ^22^ These nationally representative studies follow a common methodology and include all births (live‐born or stillborn neonates at 22 weeks of gestation or more, or weighing at least 500 g) during 1 week in all French maternity units, as described in detail elsewhere.^22^ Data for the 2021 survey were collected during 1 week in March and came from four different sources:

1. An interview with each mother in the postpartum ward, about her sociodemographic characteristics, prenatal care, and delivery.
2. Medical files, for medical conditions, complications of pregnancy, delivery, and the child's health status at birth.
3. A questionnaire completed by the head of the maternity unit, describing its principal institutional characteristics.
4. A 2‐month questionnaire completed by mothers by email or telephone, including specific questions about whether healthcare professionals requested consent for medical interventions during labor. The 2021 survey was the first to include a post‐discharge follow‐up.

The ENP 2021 was approved by the French Data Protection Authority (Commission Nationale de l'Informatique et des Liberté [CNIL], approval no.: DR‐2020‐391 on December 31, 2020), the National Council on Statistical Information (Conseil National de l'Information Statistiques [CNIS], approval no. 141/H030 on October 14, 2019), the Committee for the Protection of Persons (Comité de Protection des Personnes [CPP] on July 7, 2020), the Committee of Ethics and Scientific Research, Studies and Evaluations (Comité d'Ethique et Scientifiques pour les Recherches, les Études et les Évaluations [CESREES] on June 12, 2020) and received a label of general interest and statistical quality from the Label Committee (Visa n°2021X701SA, order of November 23, 2020). Women received detailed information about the study, including its procedures and goals, and could refuse to take part in all or part of the survey.

### 2.2 Study population

Particularities in the organization of their care led us to exclude women from overseas French districts and territories from this analysis. Analysis included women who had agreed to and did participate in the 2‐month follow‐up survey, answered the questions about consent, and had information about birth available. We included for each intervention only women whose medical files specifically mentioned its performance.

### 2.3 Failure to seek consent

The questions about consent in the 2‐month questionnaire focused on three medical interventions: oxytocin administration during labor, episiotomy, and emergency cesarean. During drafting, the questions about consent were specifically developed with the help and experience of professional societies (of anesthesiologists, midwives, obstetricians, and pediatricians) and the health‐service user association CIANE (https://ciane.net). Women were asked about the procedures they underwent during labor: “Did you have oxytocin during labor/an episiotomy/an unplanned cesarean section?” They were then asked whether their consent had been sought: “Did the midwife or doctor request your agreement to perform it?” The available answers were: “yes,” “no,” or “I don't remember.” Four groups were identified: (i) women unaware that they had received the procedure, (ii) women who knew they received it and had been asked to consent, (iii) women unable to remember whether their consent had been requested, and (iv) women reporting their consent had not been requested. Because we considered that women who were not informed of the procedure they had undergone could not have consented to it, we treated them as those who replied that their consent had not been requested: no consent sought. In the main analysis, women who reported consenting were grouped with those who did not remember as some women may have simply forgotten the request for consent. A sensitivity analysis excluded the latter group.

### 2.4 Determinants studied

Maternal characteristics included age (18–24/25–34/≥35 years), body mass index (BMI, calculated as weight in kilograms divided by the square of height in meters) (from prepregnancy weight and height, categorized as underweight or normal/overweight/obese),^23^ country of birth, education level, and expression of expectations for birth (no/orally/written birth plan). Obstetric characteristics included: history of vaginal delivery (based on parity and previous cesarean deliveries, analyzed as follows: nulliparous women/parous women with no previous vaginal birth/parous women with previous vaginal birth), multiple pregnancy, and the pregnancy's risk level, evaluated by the National Institute for Health and Care Excellence (NICE) criteria.^24^

Birth characteristics included: gestational age, mode of labor onset, cervical dilation at oxytocin initiation (0–2 cm/3–4 cm/5–9 cm/10 cm),^25^ practitioner at birth (midwife/spontaneous delivery assisted by obstetrician/instrumental vaginal delivery assisted by obstetrician), timing of cesarean (before/during labor), and time of delivery. Indications for emergency cesareans were classified by the reasons stated in the medical file. Vaginal bleeding, hypertension or pre‐eclampsia, uterine rupture and decompensation of a preexisting pathology were classified as “maternal indications”. Abnormal fetal growth or movement, chorioamnionitis, retroplacental hematoma, and oligohydramnios were classified as “fetal indications”, and other indications (e.g., stagnation of dilation, non‐engagement of the fetal head, uterine or pelvic anomalies, dystocic presentation, failed induction, etc.) as “obstetric”.

Poor neonatal outcome was defined by umbilical arterial pH <7.00 or 5‐min Apgar score <7 or neonatal resuscitation (ventilation, positive airway pressure, intubation) or admission to the neonatal intensive care unit or death in the maternity unit.

The organizational characteristics of the maternity units included level of care classified according to national regulations (III/II/I),^26^ and a variable combining legal status (public teaching hospital/other public hospital/private facility) and volume of annual births (≥2000/<2000).

### 2.5 Statistical analysis

We first described the general characteristics of the women included in the analysis and compared them with those of the women not analyzed (i.e., meeting the inclusion criteria but not responding to the question about consent). We then estimated the percentage and confidence intervals (CIs) of women reporting that their consent was not sought for each intervention. Analyses were weighted to consider the attrition bias resulting from nonresponse at 2 months so our results would be representative of births in 2021 in metropolitan France. Each woman responding to the 2‐month follow‐up was assigned a weight calculated by using homogeneous response groups^27^ to model the response probabilities at each stage of sample selection. The homogeneous response groups were constructed with the Haziza and Beaumont framework for imputation of classes and the score method.^21^, ^28^ We used Pearson's Chi^2^ test to compare percentages, Student's *t*‐test to compare means and estimated adjusted prevalence ratios (aPR) by multivariate robust variance Poisson regressions,^29^, ^30^ including candidate covariates associated in the crude analyses with a *P* value less than 0.20 or considered of interest. Poisson model allowed to control overestimation of the association strength for not rare events and aPR are interpreted as relative risk.^29^ When several variables of interest were interrelated, we introduced the most clinically relevant into the multivariate model to avoid collinearity. We performed multiple imputations with chained equations. Analyses were repeated in each dataset (15 iterations) and estimations pooled by Rubin's rules.^31^

Differences were statistically significant when *P* values were less than 0.05. The analyses used R version 4.2.1 (R Foundation for Statistical Computing), with the R “mice” version 3.16.0 and “survey” version 4.2–1 packages.

## 3 RESULTS

Among the 12 088 women with live births and available medical records, 4396 had oxytocin administered during labor (36.4%), 787 an episiotomy (8.3% of vaginal births), and 1640 women (13.6%) an emergency cesarean. Finally, the study populations included only women completing the 2‐month questionnaire: 2688 women for oxytocin administration (61.1% of those who received it), 473 women (60.1%) for episiotomy, and 946 women for emergency cesarean birth (57.7%). Figure S1 presents the populations analyzed for each intervention.

Table 1 presents the study populations' weighted characteristics. Consistently across interventions, women who received them and who answered the consent questions were older, more often nulliparous and born in France, and more likely to have expressed their expectations for the birth (Tables S1–S3).

**TABLE 1:** Description of the characteristics of the women, deliveries, neonates, and maternity units of the study populations. ENP 2021, Metropolitan France.

	Oxytocin (*N* = 2688)	Episiotomy (*N* = 473)	Cesarean birth (*N* = 946)
	% (95% CI), weighted	% (95% CI), weighted	% (95% CI), weighted
*Mother*			
Maternal age (years), mean ± SD	30.5 ± 5.2	29.6 ± 5.2	31.1 ± 5.6
<24	12.7 (11.3–14.0)	15.4 (11.7–20.0)	12.0 (9.7–15.0)
25–34	65.6 (63.6–68.0)	68.1 (63.2–73.0)	59.8 (56.4–63.0)
≥35	21.7 (20.1–23.0)	16.4 (13.1–20.0)	28.1 (25.0–31.0)
Maternal BMI (kg/m^2^), mean ± SD	25.1 ± 5.5	23.2 ± 4.1	26.2 ± 6.3
<25	59.9 (57.8–62.0)	77.3 (73.2–81.0)	53.0 (49.5–56.0)
25–30	23.2 (21.5–25.0)	16.3 (13.2–20.0)	23.8 (21.0–27.0)
≥30	16.9 (15.5–18.0)	6.4 (4.5–9.0)	23.3 (20.4–26.0)
History of vaginal birth			
Parous with a history of vaginal birth	38.2 (36.1–40.0)	14.5 (11.3–18.0)	24.7 (21.6–28.0)
Nulliparous	56.0 (53.9–58.0)	80.2 (76.0–84.0)	58.7 (55.1–62.0)
Parous with no previous vaginal birth	5.7 (4.9–7.0)	5.3 (3.6–8.0)	16.6 (14.2–19.0)
Country of birth			
France	77.8 (75.8–80.0)	79.4 (74.5–84.0)	74.4 (70.8–78.0)
Abroad	22.2 (20.2–24.0)	20.6 (16.5–26.0)	25.6 (22.3–29.0)
Education level			
High school	42.2 (40.1–44.0)	31.5 (26.9–37.0)	42.6 (39.1–46.0)
1–2 years post‐secondary education	16.9 (15.5–18.0)	13.4 (10.6–17.0)	19.6 (17.1–22.0)
3–4 years post‐secondary education	18.4 (17.0–20.0)	23.3 (19.6–27.0)	19.4 (17.0–22.0)
≥5 years post‐secondary education	22.5 (21.0–24.0)	31.8 (27.6–36.0)	18.4 (15.9–21.0)
Expression of expectations for birth			
No	69.4 (67.5–71.0)	68.9 (64.3–73.0)	71.7 (68.6–75.0)
Written birth plan	10.8 (9.7–12.0)	13.3 (10.6–17.0)	10.4 (8.7–12.0)
Orally expressed expectations	19.7 (18.1–22.0)	17.9 (14.5–22.0)	17.8 (15.4–21.0)
Low risk pregnancy			
Yes	53.9 (51.8–56.0)	65.1 (60.3–70.0)	33.7 (30.7–37.0)
No	46.1 (44.0–48.0)	34.9 (30.4–40.0)	66.3 (63.0–69.0)
Multiple pregnancy			
No	98.7 (97.9–99.0)	99.8 (98.3–99.9)	96.4 (94.8–98.0)
Yes	1.3 (0.1–2.0)	0.2 (0.1–0.2)	3.6 (2.4–5.0)
*Child*			
Gestational age at birth (weeks)			
<37	4.4 (3.5–6.0)	2.6 (1.5–4.0)	14.8 (12.3–18.0)
≥37	95.6 (94.4–97.0)	97.4 (95.6–99.0)	85.2 (82.4–88.0)
Poor neonatal outcome			
No	83.3 (81.4–85.0)	85.0 (80.8–88.0)	64.6 (60.8–68.0)
Yes	16.7 (15.0–19.0)	15.0 (11.6–19.0)	35.4 (31.8–39.0)
*Facility*			
Level of care			
I	21.7 (20.1–23.0)	22.5 (18.8–27.0)	21.5 (18.9–24.0)
IIa and IIb	52.1 (50.0–54.0)	50.8 (45.9–56.0)	50.1 (46.6–54.0)
III	26.2 (24.4–28.0)	26.7 (22.4–31.0)	28.4 (25.4–32.0)
Maternity unit legal status and volume (births/year)			
Public teaching hospital	18.9 (17.3–21.0)	18.9 (15.3–23.0)	21.7 (18.9–25.0)
Other public^a^ ≥2000	26.5 (24.6–28.0)	25.9 (21.7–30.0)	26.8 (23.7–30.0)
Other public^a^ <2000	31.5 (29.7–33.0)	35.1 (30.7–40.0)	31.7 (28.6–35.0)
Private ≥2000	6.6 (5.7–8.0)	4.4 (2.9–7.0)	5.1 (3.8–7.0)
Private <2000	16.5 (15.1–18.0)	15.7 (12.6–19.0)	14.7 (12.6–17.0)

Abbreviations: BMI, body mass index; CI, confidence interval; NA, not available; SD, standard deviation.

^a^ Other public category includes public facilities (except for teaching hospitals) and private non‐profit facilities performing public service.

Among the women analyzed, 44.7% (weighted, 95% CI: 42.6–47.0; *n* = 1162) reported that their consent had not been requested for oxytocin administration (including 816 women unaware they received it), 60.2% (weighted, 95% CI: 55.4–65.0; *n* = 280) for episiotomy (including 25 unaware of the procedure), and 36.6% (weighted, 95% CI: 33.3–40.0; *n* = 343) for emergency cesarean birth.

In univariate analyses, the factors associated with the absence of consent varied between interventions. They were most often mother‐related for oxytocin administration, organizational for episiotomy, and related to the birth characteristics for cesareans (Table S4).

**FIGURE 1:**
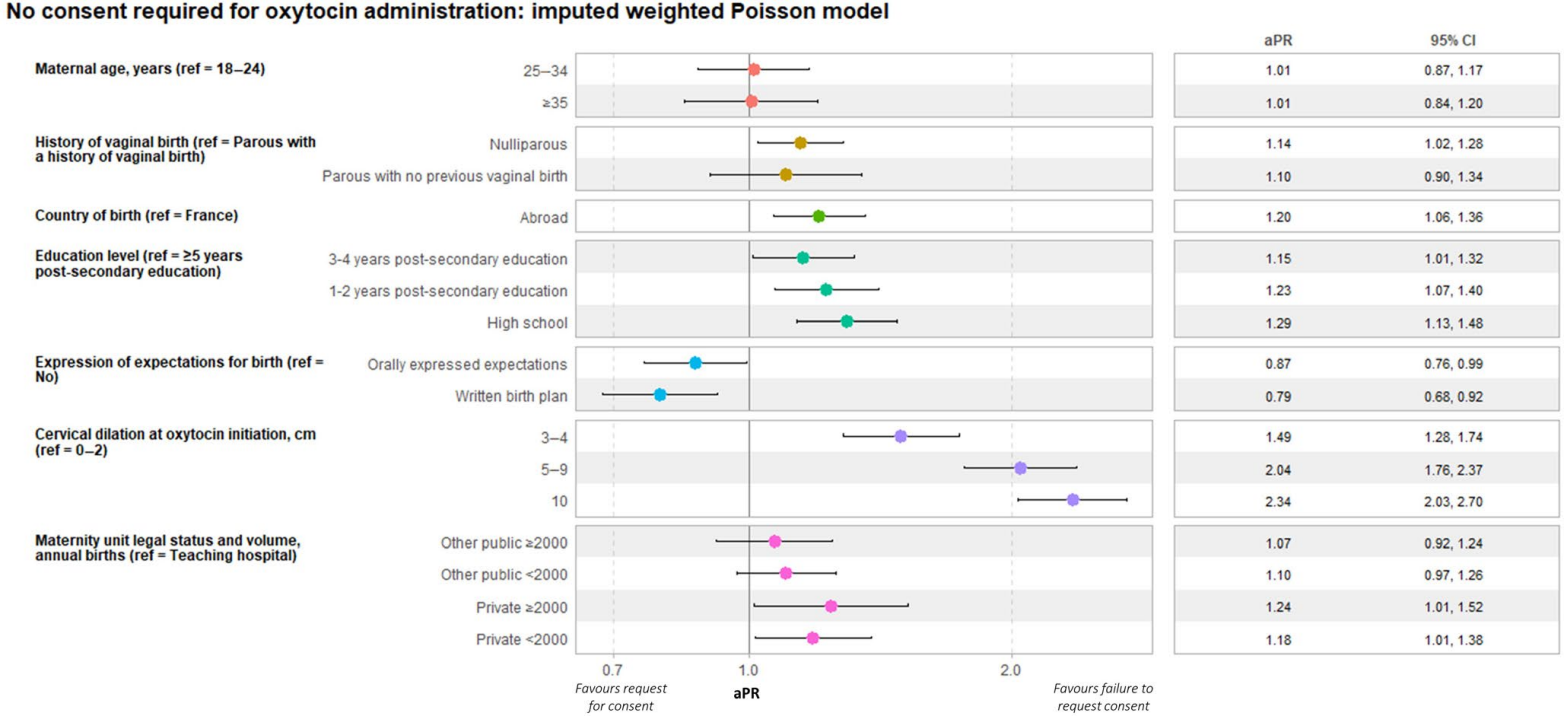
Association of women's sociodemographic characteristics, pregnancy, delivery, neonatal, and maternity characteristics with request for consent for oxytocin administration (not including prophylactic oxytocin dose at delivery of shoulder). ENP 2021, Metropolitan France, *N* = 2688 Poisson regression, weighted and imputed data. Other public category includes public facilities (except for teaching hospitals) and private non‐profit facilities performing public service. aPR, adjusted prevalence ratio; CI, confidence interval; ref, reference.

In the weighted multivariable analysis, lack of consent for oxytocin administration during labor was reported more frequently by nulliparous (aPR 1.14; 95% CI: 1.02–1.28) than by parous women with a previous vaginal birth, by women born abroad (aPR 1.20; 95% CI: 1.06–1.36 vs born in France), and by women with an education level below 5 years of post‐secondary studies (aPR 1.15; 95% CI: 1.01–1.32 for those with 3–4 years of post‐secondary education: aPR 1.23; 95% CI: 1.07–1.40 for those with 1–2 years of post‐secondary education and aPR 1.29; 95% CI: 1.13–1.48 for those who attended some—or completed—high school). Conversely, women who orally expressed expectations for childbirth (aPR 0.87; 95% CI: 0.76–0.99) or had a written birth plan (aPR 0.79; 95% CI: 0.68–0.92) reported lack of consent less often than women who did not. More advanced cervical dilation at oxytocin initiation was associated with an absence of consent (aPR 1.49; 95% CI: 1.28–1.74 at 3–4 cm; aPR 2.04; 95% CI: 1.76–2.37 at 5–9 cm, and aPR 2.34; 95% CI: 2.03–2.70 at full dilation, compared with less than 3 cm). Consent was sought less frequently in private maternity units than in teaching hospitals, for units with 2000 or more deliveries annually (aPR 1.24; 95% CI: 1.01–1.52) and units with <2000 (aPR 1.18; 95% CI: 1.01–1.38) (Figure 1).

**FIGURE 2:**
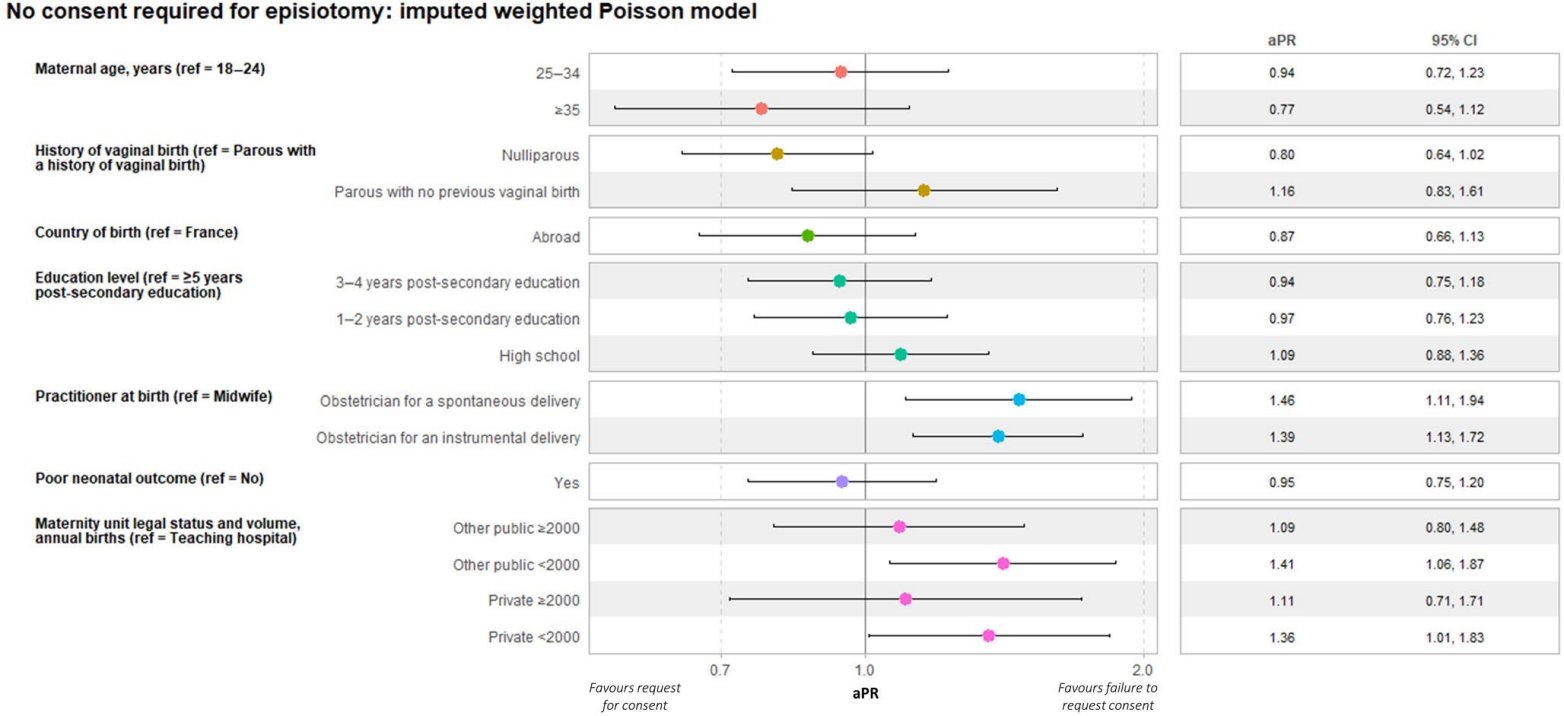
Association of women's sociodemographic characteristics, delivery, neonatal, and maternity characteristics with request for consent to episiotomy. ENP 2021, Metropolitan France, *N* = 473. Poisson regression, weighted and imputed data. Other public category includes public facilities (except for teaching hospitals) and private nonprofit facilities performing public service. aPR, adjusted prevalence ratio; CI, confidence interval; ref, reference.

Consent for episiotomy was requested less often from women with an obstetrician‐assisted spontaneous vaginal (aPR 1.46; 95% CI: 1.11–1.94) or instrumental (aPR 1.39; 95% CI: 1.13–1.72) birth, compared with midwife‐assisted spontaneous birth, and from those giving birth in maternity units with fewer than 2000 annual deliveries in either public (aPR 1.41; 95% CI: 1.06–1.87) or private (aPR 1.36; 95% CI: 1.01–1.83) compared with teaching hospitals. No association was identified with maternal or neonatal characteristics (Figure 2).

**FIGURE 3:**
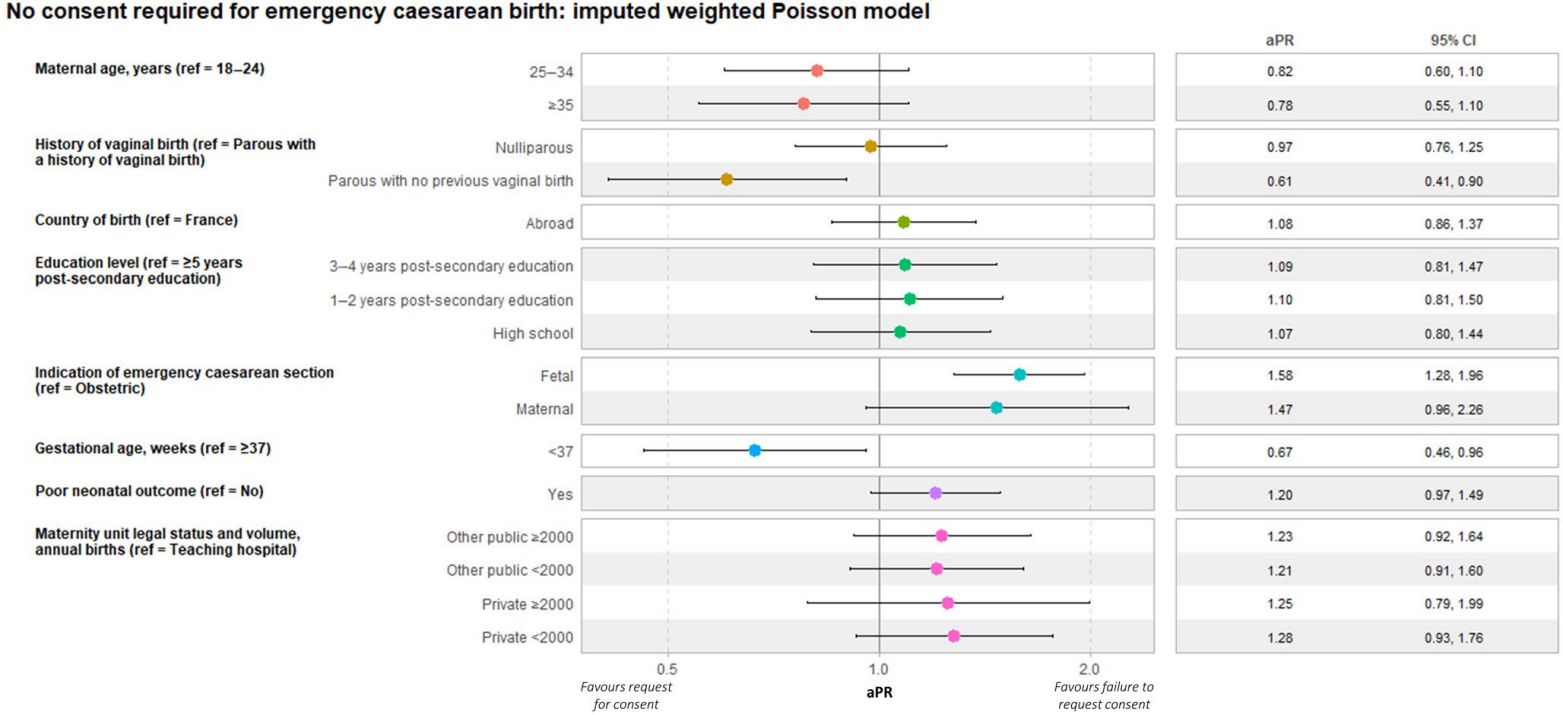
Association of women's sociodemographic characteristics, delivery, neonatal, and maternity unit characteristics with request for consent to emergency cesarean birth. ENP 2021, Metropolitan France, *N* = 946. Poisson regression, weighted and imputed data. Other public category includes public facilities (except for teaching hospitals) and private non‐profit facilities performing public service. aPR, adjusted prevalence ratio; CI, confidence interval; ref, reference.

Failure to seek consent for emergency cesarean births was less frequent in parous women without versus with a previous vaginal birth (aPR 0.61; 95% CI: 0.41–0.90), and in births before versus after 37 weeks (aPR 0.67; 95% CI: 0.46–0.96). Professionals failed to seek consent more often in cesareans for fetal compared with obstetric indications (aPR 1.58; 95% CI: 1.28–1.96) (Figure 3).

The sensitivity analyses excluding women who could not remember whether their consent had been sought yielded similar results (results not shown).

## 4 DISCUSSION

In this nationwide population‐based survey, women frequently reported that clinicians failed to request their consent for medical interventions during childbirth; these rates ranged from 36.6% for emergency cesarean delivery to 44.7% for oxytocin administration and 60.2% for episiotomy. The identification of factors associated with lack of consent suggests that women's involvement in medical decisions can frequently be improved. For oxytocin administration, this rate could be improved by increasing staff attention to each woman's expectations during labor. Improving obstetric practices could enhance consent to episiotomy, and particular attention to consent to emergency cesareans is essential.

Our study found high rates of interventions for which consent was not sought, indicating not only insufficient consent requirement in obstetrics but also likely dissatisfaction among women with the consent process as carried out.^32^ These rates were consistently higher than those previously reported by women in studies carried out in HICs, whatever the intervention studied.^10^, ^11^, ^13^, ^16^, ^17^, ^18^ However, they were mainly self‐reported, suffering from a possible selection and classification bias for women unaware of the intervention they received. Only one study conducted by a French health‐service users' association among volunteers reported a higher rate of lack of consent for episiotomy.^20^ Recent debates about mistreatment in healthcare in France may have led to improving practices, but there is still room for amelioration.^33^ Identifying determinants related to the absence of consent should thus be a major step forward in changing practices further.

Regarding maternal characteristics, we found that consent was requested less frequently from women born abroad, but this only applied to consent for oxytocin administration. Some differential perinatal care by maternal ethnicity or nationality has been described in France and in other HICs, especially in the USA.^13^, ^33^, ^34^, ^35^, ^36^ Women with lower education levels were less likely to be asked to consent to oxytocin, possibly because healthcare professionals do not provide comprehensive information to women they perceive as less likely to grasp the implications.^37^, ^38^ To limit differential care based on women's characteristics, raising health professionals' awareness on this issue is essential, as it frequently results from implicit biases.^3^, ^35^ Conversely, irrespective of the other socioeconomic characteristics studied (maternal age, level of education and country of birth), women with a birth plan were more likely to be asked to consent to oxytocin administration. Birth plans are designed as a tool to facilitate communication and promote women's participation in intrapartum decision making and may call staff's attention to the systematically seeking of consent.^39^ Although writing a birth plan is becoming more common, it remains limited to women with specific profiles.^21^, ^40^ Promoting the drafting of birth plans is a key factor in empowering women. Finally, nulliparas reported their consent to oxytocin administration was requested less frequently than parous women, as previously reported.^19^ Women with previous cesarean births reported less frequent lack of consent for emergency cesareans than first‐timers. Understanding the obstetric situation may be easier for women who have already experienced it.

Regarding obstetric characteristics, the frequency of consent requests for oxytocin administration gradually decreased as labor progressed, indicating a potential decrease in professionals' vigilance about consent once active labor is underway. Delivery assisted by an obstetrician, rather than by a midwife, was associated with a higher rate of failure to seek consent for episiotomy, aligning with similar findings in the USA.^13^ This association may stem from varying levels of awareness regarding consent issues among professions, but interpretation is constrained by the lack of indications for episiotomy. Finally, the lack of request for consent to emergency cesarean birth appeared to be related to its context. We found it was more frequent for fetal than obstetric indications. This variable imperfectly approximates the degree of emergency but suggests an acute event. Consent for emergency cesarean section is a hotly debated topic among healthcare providers and lawyers and raises questions about the available time for information and consent in life‐threatening emergencies.^41^, ^42^, ^43^ Factors linked to the organization of care could be substantially improved by modifying some department policies. Among others, adding “obtained consent” items for each intervention to medical record forms might encourage professionals to request it routinely. Explanation of the care provided during childbirth could become systematic on entry to the delivery room, especially if a dedicated caregiver responsible for providing information and requesting consent in emergency situations is designated. Finally, simulation‐based professional training could be valuable for developing and practicing appropriate methods for seeking informed consent.^44^

Overall, although the three evaluated procedures are all among the most routinely performed during childbirth, different determinants of the failure to seek consent were identified for each of them. This suggests that professionals' perception of risk can lead to differences in the shared decision making process, sometimes compounded by distinctions based on women's individual characteristics.

Requesting women's consent for all interventions performed during childbirth is not optional. Not only are information and seeking consent legal obligations, but women's involvement in decision making is also a major element in improving their childbirth experience.^45^, ^46^, ^47^ The absence of information and consent can be experienced as the unilateral management of care by professionals and can lead women to feel excluded from their child's birth and dispossessed of their own bodies.^4^

### 4.1 Strengths and limitations

The ENP 2021 is the first of this survey series to examine consent‐seeking practices in France and the first population‐based representative study we know of on this important topic in HICs. Our results are strengthened by the availability of the women's medical files, enabling us to focus specifically on the experiences of women who actually underwent the procedures and to identify women unaware of the procedures they had received. The quality of the birth data, collected by trained midwives, and the response rate at 2 months were both high.^22^ Nonetheless, our analyses may have some limitations. First, only women who underwent the intervention were included in these analyses, excluding rare situations where women refused an intervention to which they were asked to consent.^48^ Our results should not be interpreted to assess if women consented to these procedures but rather as evaluating their assessment of perinatal professionals' consent‐seeking practices. Furthermore, we evaluated women's retrospective perception of consent seeking 2 months post‐intervention, acknowledging the potential for memory bias. Nevertheless, we hypothesized that women who kept a feeling of inclusion in the decision making process, even without explicit memory of the consent request, would be more likely to respond affirmatively that they consent was sought, or express uncertainty rather than negation. We pooled women who responded that they did not remember (5–6%) with those who reported they consent was requested. Finally, despite the high response rate at the 2‐month questionnaire, attrition was stronger among women at lower socioeconomic levels, as previously described in birth cohorts.^49^ However, the available data about the women who did not respond allowed us to weight the analyses.

A great deal of research remains to be done to better understand women's expectations about how their consent should be sought (wording, timing, provider, duration, etc.), so mixed‐method studies should be useful. The mechanisms involved in disrupting the professional‐patient relationship have yet to be explored. Observations of professionals' practices would shed light on the current methods of seeking consent and limiting factors.^50^

## 5 CONCLUSION

Failure to seek consent for interventions during childbirth was frequent in France. The main determinants associated with it were related to women's characteristics, obstetric emergencies, staff practices, and organizational factors. Dissemination of these results to staff will raise awareness of opportunities for significant improvement. Professionals should have specific training in initial and continuing education, emphasizing the importance of seeking consent for all interventions.

## AUTHOR CONTRIBUTIONS

Anne Evrard, Nathalie Lelong, and Camille Le Ray designed the study. Marianne Jacques carried out the statistical analysis and wrote the first draft of the manuscript. Camille Le Ray is the scientific coordinator of the ENP 2021, and Nathalie Lelong is the co‐coordinator. Camille Le Ray made significant revisions to the first draft; all authors revised successive versions of the article and approved the final article.

## FUNDING INFORMATION

The 2021 National Perinatal Survey was funded by the French Ministry of Health (DREES, DGS, DGOS) and the French National Public Health Agency (Santé Publique France). MJ received funding from the Université Paris Cité – Paris Cité University – for her PhD and this research was supported by a grant from the Mustela Foundation. These funders had no role in the study design, data collection and analysis, decision to publish, or preparation of the manuscript.

## CONFLICT OF INTEREST STATEMENT

The authors have no conflicts of interest or disclosures to declare.

## Supporting information


Appendix S1:

## Data Availability

Data sharing is not applicable to this article as no new data were created or analyzed in this study.
