# Supplementary material for: Consent for interventions during childbirth: A national population‐based study
Source: Int J Gynaecol Obstet. 2024 Aug 2;168(1):333–42. doi: 10.1002/ijgo.15830 (PMC11649875; doi:10.1002/ijgo.15830)
Supplement: Supplementary file 1 — Appendix S1: [file IJGO-168-333-s001.docx]

**Consent for interventions during childbirth: a national population-based study**

# APPENDICES

## Figure S1. Flow chart

**Table S1.** Comparison of the characteristics of the women, deliveries, neonates, and maternity units of the women whose data were and were not analyzed, among women who had oxytocin administered.

**Table S2.** Comparison of the characteristics of the women, deliveries, neonates, and maternity units of the women whose data were and were not analyzed, among the women who had an episiotomy.

**Table S3.** Comparison of the characteristics of the women, deliveries, neonates, and maternity units of the women whose data were and were not analyzed, among the women who had an emergency cesarean birth.

**Table S4.** Comparison of characteristics of women, deliveries, children, and maternity units for women whose consent was and was not requested.


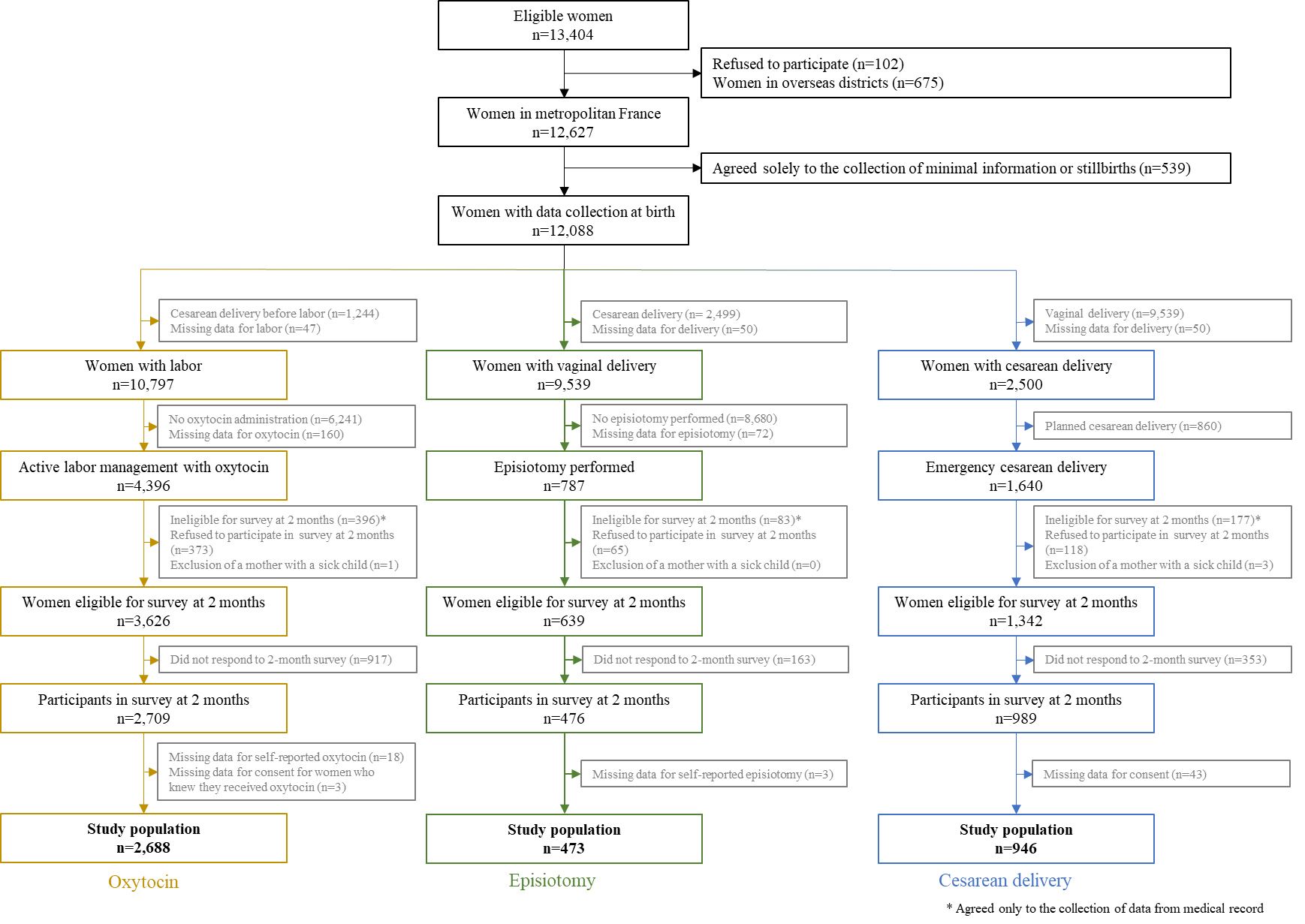


## **Figure S1.** Flow chart

## **Table S1.** Comparison of the characteristics of the women, deliveries, neonates, and maternity units of the women whose data were and were not analyzed, among women with oxytocin administered. *ENP* 2021, Metropolitan France, N=4,396.

|  | Not analyzed  n=1,708 | Analyzed  n=2,688 | *P*-value |
| --- | --- | --- | --- |
|  | n (%) | n (%) |  |
| **Mother** |  |  |  |
| Maternal age (years), mean ± SD | 29.9 ± 5.6 | 30.8 ± 5.1 | **<.001** |
| <24 | 298 (17.5) | 280 (10.4) | **<.001** |
| [25-34] | 1,054 (61.8) | 1,795 (66.8) |  |
| ≥35 | 354 (20.8) | 613 (22.8) |  |
| *NA* | *2* | *0* |  |
| Maternal BMI (kg/m²), mean ± SD | 25.1 ± 5.3 | 25.0 ± 5.5 | .522 |
| <25 | 729 (57.2) | 1,617 (60.5) | .116 |
| [25–30[ | 327 (25.7) | 616 (23.1) |  |
| ≥30 | 218 (17.1) | 438 (16.4) |  |
| *NA* | *434* | *17* |  |
| History of vaginal birth |  |  | **<.001** |
| Parous with a history of vaginal birth | 753 (44.1) | 928 (34.5) |  |
| Nulliparous | 850 (49.8) | 1,609 (59.9) |  |
| Parous with no previous vaginal birth | 104 (6.1) | 149 (5.5) |  |
| *NA* | *1* | *2* |  |
| Country of birth |  |  | **<.001** |
| France | 912 (70.1) | 2,196 (82.5) |  |
| Abroad | 389 (29.9) | 465 (17.5) |  |
| *NA* | *407* | *27* |  |
| Education level |  |  | **<.001** |
| High school | 678 (51.9) | 938 (34.9) |  |
| 1-2 years post-secondary education | 228 (17.4) | 484 (18.0) |  |
| 3-4 years post-secondary education | 200 (15.3) | 552 (20.5) |  |
| ≥ 5 years post-secondary education | 201 (15.4) | 713 (26.5) |  |
| *NA* | *401* | *1* |  |
| Expression of expectations for birth |  |  | **<.001** |
| No | 999 (76.8) | 1,795 (66.8) |  |
| Written birth plan | 95 (7.3) | 339 (12.6) |  |
| Orally expressed expectations | 207 (15.9) | 553 (20.6) |  |
| *NA* | *407* | *1* |  |
| Low risk pregnancy |  |  | .203 |
| Yes | 915 (53.6) | 1,494 (55.6) |  |
| No | 793 (46.4) | 1,194 (44.4) |  |
| Multiple pregnancy |  |  | **.002** |
| No | 1,669 (97.7) | 2,659 (98.9) |  |
| Yes | 39 (2.3) | 29 (1.1) |  |
| **Child** |  |  |  |
| Gestational age at birth (weeks) |  |  | .615 |
| <37 | 61 (3.6) | 104 (3.9) |  |
| ≥37 | 1,646 (96.4) | 2,584 (96.1) |  |
| *NA* | *1* | *0* |  |
| Poor neonatal outcome |  |  | **.024** |
| No | 1,159 (81.8) | 1,946 (84.7) |  |
| Yes | 258 (18.2) | 352 (15.3) |  |
| *NA* | *291* | *390* |  |
| **Facility** |  |  |  |
| Level of care |  |  | .072 |
| I | 326 (19.1) | 590 (21.9) |  |
| IIa and IIb | 922 (54.0) | 1,408 (52.4) |  |
| III | 460 (26.9) | 690 (25.7) |  |
| Maternity unit legal status and volume (births/year) |  |  | **.010** |
| Public teaching hospital | 343 (20.1) | 504 (18.8) |  |
| Other public* ≥2000 | 433 (25.4) | 701 (26.1) |  |
| Other public* <2000 | 515 (30.2) | 863 (32.1) |  |
| Private ≥2000 | 161 (9.4) | 181 (6.7) |  |
| Private <2000 | 256 (15.0) | 439 (16.3) |  |

*BMI: body mass index, NA: not available, SD: standard deviation*

° Not including prophylactic oxytocin dose at delivery of shoulder

* Other public category includes public facilities (except for teaching hospitals) and private nonprofit facilities performing public service.

Chi-square test for qualitative data, Student t test for quantitative data

## **Table S2.** Comparison of the characteristics of the women, deliveries, neonates, and maternity units of the women whose data were and were not analyzed, among the women who had an episiotomy. *ENP* 2021, Metropolitan France, N=787.

|  | Not analyzed  n=314 | Analyzed  n=473 | *P*-value |
| --- | --- | --- | --- |
|  | n (%) | n (%) |  |
| **Mother** |  |  |  |
| Maternal age (years), mean ± SD | 28.5 ± 5.1 | 30.2 ± 4.8 | **<.001** |
| ≤24 | 77 (24.5) | 53 (11.2) | **<.001** |
| [25-34] | 201 (64.0) | 337 (71.2) |  |
| ≥35 | 36 (11.5) | 83 (17.5) |  |
| Maternal BMI (kg/m²), mean ± SD | 23.3 ± 4.3 | 23.2 ± 4.1 | .737 |
| <25 | 160 (71.1) | 358 (76.2) | .286 |
| [25–30[ | 44 (19.6) | 81 (17.2) |  |
| ≥30 | 21 (9.3) | 31 (6.6) |  |
| *NA* | *89* | *3* |  |
| History of vaginal birth |  |  | .512 |
| Parous with a history of vaginal birth | 54 (17.2) | 67 (14.2) |  |
| Nulliparous | 243 (77.4) | 380 (80.3) |  |
| Parous with no previous vaginal birth | 17 (5.4) | 26 (5.5) |  |
| Country of birth |  |  | **<.001** |
| France | 155 (67.7) | 393 (83.8) |  |
| Abroad | 74 (32.3) | 76 (16.2) |  |
| *NA* | *85* | *4* |  |
| Education level |  |  | **<.001** |
| High school | 113 (48.9) | 121 (25.6) |  |
| 1-2 years post-secondary education | 42 (18.2) | 67 (14.2) |  |
| 3-4 years post-secondary education | 34 (14.7) | 117 (24.7) |  |
| ≥ 5 years post-secondary education | 42 (18.2) | 168 (35.5) |  |
| *NA* | *83* | *0* |  |
| Expression of expectations for birth |  |  | .131 |
| No | 166 (73.1) | 316 (66.8) |  |
| Written birth plan | 22 (9.7) | 70 (14.8) |  |
| Orally expressed expectations | 39 (17.2) | 87 (18.4) |  |
| *NA* | *87* | *0* |  |
| Low risk pregnancy |  |  | >.99 |
| Yes | 203 (64.6) | 305 (64.5) |  |
| No | 111 (35.4) | 168 (35.5) |  |
| Multiple pregnancy |  |  | NC |
| No | 314 (100.0) | 472 (99.8) |  |
| Yes | 0 (0.0) | 1 (0.2) |  |
| **Child** |  |  |  |
| Gestational age at birth (weeks) |  |  | .288 |
| <37 | 5 (1.6) | 13 (2.7) |  |
| ≥37 | 309 (98.4) | 460 (97.3) |  |
| Poor neonatal outcome |  |  | .059 |
| No | 209 (80.1) | 346 (85.6) |  |
| Yes | 52 (19.9) | 58 (14.4) |  |
| *NA* | *53* | *69* |  |
| **Facility** |  |  |  |
| Level of care |  |  | .759 |
| I | 76 (24.2) | 107 (22.6) |  |
| IIa and IIb | 163 (51.9) | 243 (51.4) |  |
| III | 75 (23.9) | 123 (26.0) |  |
| Maternity unit legal status and volume (deliveries/year) |  |  | .193 |
| Public teaching hospital | 41 (13.1) | 87 (18.4) |  |
| Other public* ≥2000 | 88 (28.0) | 121 (25.6) |  |
| Other public* <2000 | 115 (36.6) | 169 (35.7) |  |
| Private ≥2000 | 22 (7.0) | 21 (4.4) |  |
| Private <2000 | 48 (15.3) | 75 (15.9) |  |

*BMI: body mass index, NA: not available, NC: not calculated, SD: standard deviation*

* Other public category includes public facilities (except for teaching hospitals) and private nonprofit facilities performing public service.

Chi-square test for qualitative data, Student t test for quantitative data

## **Table S3.** Comparison of the characteristics of the women, deliveries, neonates, and maternity units of the women whose data were and were not analyzed, among the women who had an emergency cesarean birth. *ENP* 2021, Metropolitan France, N=1,640.

|  | Not analyzed  n=694 | Analyzed  n=946 | *P*-value |
| --- | --- | --- | --- |
|  | n (%) | n (%) |  |
| **Mother** |  |  |  |
| Maternal age (years), mean ± SD | 31.4 ± 6.1 | 31.4 ± 5.4 | .898 |
| ≤24 | 96 (13.9) | 88 (9.3) | **.008** |
| [25-34] | 385 (55.7) | 578 (61.1) |  |
| ≥35 | 210 (30.4) | 280 (29.6) |  |
| *NA* | *3* | *0* |  |
| Maternal BMI (kg/m²), mean ± SD | 25.8 ± 5.3 | 26.0 ± 6.1 | .548 |
| <25 | 258 (51.9) | 509 (54.4) | .645 |
| [25–30[ | 127 (25.6) | 222 (23.7) |  |
| ≥30 | 112 (22.5) | 205 (21.9) |  |
| *NA* | *197* | *10* |  |
| History of vaginal birth |  |  | **<.001** |
| Parous with a history of vaginal birth | 201 (29.0) | 195 (20.7) |  |
| Nulliparous | 347 (50.0) | 595 (63.0) |  |
| Parous with no previous vaginal birth | 146 (21.0) | 154 (16.3) |  |
| *NA* | *0* | *2* |  |
| Country of birth |  |  | **<.001** |
| France | 324 (63.2) | 743 (79.5) |  |
| Abroad | 189 (36.8) | 192 (20.5) |  |
| *NA* | *181* | *11* |  |
| Education level |  |  | **<.001** |
| High school | 270 (52.6) | 340 (36.0) |  |
| 1-2 years post-secondary education | 96 (18.7) | 196 (20.7) |  |
| 3-4 years post-secondary education | 68 (13.3) | 208 (22.0) |  |
| ≥ 5 years post-secondary education | 79 (15.4) | 201 (21.3) |  |
| *NA* | *181* | *1* |  |
| Expression of expectations for birth |  |  | **<.001** |
| No | 405 (79.3) | 653 (69.1) |  |
| Written birth plan | 33 (6.5) | 116 (12.3) |  |
| Orally expressed expectations | 73 (14.3) | 176 (18.6) |  |
| *NA* | *183* | *1* |  |
| Low risk pregnancy |  |  | **.008** |
| Yes | 213 (30.7) | 350 (37.0) |  |
| No | 481 (69.3) | 596 (63.0) |  |
| Multiple pregnancy |  |  | **.007** |
| No | 654 (94.2) | 917 (96.9) |  |
| Yes | 40 (5.8) | 29 (3.1) |  |
| **Child** |  |  |  |
| Gestational age at birth (weeks) |  |  | **<.001** |
| <37 | 151 (21.8) | 117 (12.4) |  |
| ≥37 | 542 (78.2) | 829 (87.6) |  |
| *NA* | *1* | *0* |  |
| Poor neonatal outcome |  |  | **<.001** |
| No | 342 (58.3) | 560 (67.2) |  |
| Yes | 245 (41.7) | 273 (32.8) |  |
| *NA* | *107* | *113* |  |
| **Facility** |  |  |  |
| Level of care |  |  | **<.001** |
| I | 114 (16.4) | 216 (22.8) |  |
| IIa and IIb | 340 (49.0) | 473 (50.0) |  |
| III | 240 (34.6) | 257 (27.2) |  |
| Maternity unit legal status and volume (births/year) |  |  | **.023** |
| Public teaching hospital | 166 (23.9) | 194 (20.5) |  |
| Other public* ≥2000 | 194 (28.0) | 247 (26.1) |  |
| Other public* <2000 | 199 (28.7) | 307 (32.5) |  |
| Private ≥2000 | 51 (7.3) | 49 (5.2) |  |
| Private <2000 | 84 (12.1) | 149 (15.8) |  |

*BMI: body mass index, NA: not available, SD: standard deviation*

* Other public category includes public facilities (except for teaching hospitals) and private nonprofit facilities performing public service.

Chi-square test for qualitative data, Student t test for quantitative data

## Table S4. Comparison of characteristics of women, deliveries, children, and maternity units for women whose consent was and was not requested.

|  | Oxytocin°  (n=2,688) | | | | Episiotomy  (n=473) | | | | Emergency cesarean birth  (n=946) | | |
| --- | --- | --- | --- | --- | --- | --- | --- | --- | --- | --- | --- |
|  | CR  n=1,526 | NCR  n=1,162 | *P*-value | CR  n=193 | | NCR  n=280 | *P*-value | CR  n=603 | | NCR  n=343 | *P*-value |
|  | n (%) | n (%) |  | n (%) | | n (%) |  | n (%) | | n (%) |  |
| **Mother and pregnancy** |  |  |  |  | |  |  |  | |  |  |
| Maternal age (years) |  |  | **.015** |  | |  | .423 |  | |  | .289 |
| <24 | 139 (49.6) | 141 (50.4) |  | 23 (43.4) | | 30 (56.6) |  | 50 (56.8) | | 38 (43.2) |  |
| [25-34] | 1,019 (56.8) | 776 (43.2) |  | 131 (38.9) | | 206 (61.1) |  | 368 (63.7) | | 210 (36.3) |  |
| ≥35 | 368 (60.0) | 245 (40.0) |  | 39 (47.0) | | 44 (53.0) |  | 185 (66.1) | | 95 (33.9) |  |
| Maternal BMI (kg/m²) |  |  | .601 |  | |  | .831 |  | |  | .973 |
| <25 | 910 (56.3) | 707 (43.7) |  | 149 (41.6) | | 209 (58.4) |  | 324 (63.7) | | 185 (36.3) |  |
| [25–30[ | 347 (56.3) | 269 (43.7) |  | 31 (38.3) | | 50 (61.7) |  | 143 (64.4) | | 79 (35.6) |  |
| ≥30 | 258 (58.9) | 180 (41.1) |  | 12 (38.7) | | 19 (61.3) |  | 130 (63.4) | | 75 (36.6) |  |
| *NA* | *11* | *6* |  | *1* | | *2* |  | *6* | | *4* |  |
| History of vaginal birth |  |  | **<.001** |  | |  | .430 |  | |  | **<.001** |
| Parous with a history of vaginal birth | 586 (63.1) | 342 (36.9) |  | 25 (37.3) | | 42 (62.7) |  | 120 (61.5) | | 75 (38.5) |  |
| Nulliparous | 855 (53.1) | 754 (46.9) |  | 160 (42.1) | | 220 (57.9) |  | 360 (60.5) | | 235 (39.5) |  |
| Parous with no previous vaginal birth | 83 (55.7) | 66 (44.3) |  | 8 (30.8) | | 18 (69.2) |  | 123 (79.9) | | 31 (20.1) |  |
| *NA* | *2* | *0* |  | *0* | | *0* |  | *0* | | *2* |  |
| Country of birth |  |  | **.006** |  | |  | .0104 |  | |  | .777 |
| France | 1,273 (58.0) | 923 (42.0) |  | 154 (39.2) | | 239 (60.8) |  | 475 (63.9) | | 268 (36.1) |  |
| Abroad | 237 (51.0) | 228 (49.0) |  | 38 (50.0) | | 38 (50.0) |  | 120 (62.5) | | 72 (37.5) |  |
| *NA* | *16* | *11* |  | *1* | | *3* |  | *8* | | *3* |  |
| Education level |  |  | **.010** |  | |  | .577 |  | |  | .863 |
| High school | 506 (53.9) | 432 (46.1) |  | 44 (36.4) | | 77 (63.6) |  | 214 (62.9) | | 126 (37.1) |  |
| 1-2 years post-secondary education | 262 (54.1) | 222 (45.9) |  | 27 (40.3) | | 40 (59.7) |  | 125 (63.8) | | 71 (36.2) |  |
| 3-4 years post-secondary education | 319 (57.8) | 233 (42.2) |  | 53 (45.3) | | 64 (54.7) |  | 130 (62.5) | | 78 (37.5) |  |
| ≥ 5 years post-secondary education | 439 (61.6) | 274 (38.4) |  | 69 (41.1) | | 99 (58.9) |  | 133 (66.2) | | 68 (33.8) |  |
| *NA* | *0* | *1* |  | *0* | | *0* |  | *1* | | *0* |  |
| Expression of expectations for birth |  |  | **<.001** |  | |  | .502 |  | |  | .934 |
| No | 970 (54.0) | 825 (46.0) |  | 125 (39.6) | | 191 (60.4) |  | 418 (64.0) | | 235 (36.0) |  |
| Written birth plan | 214 (63.1) | 125 (36.9) |  | 33 (47.1) | | 37 (52.9) |  | 74 (63.8) | | 42 (36.2) |  |
| Orally expressed expectations | 342 (61.8) | 211 (38.2) |  | 35 (40.2) | | 52 (59.8) |  | 110 (62.5) | | 66 (37.5) |  |
| *NA* | *0* | *1* |  | *0* | | *0* |  | *1* | | *0* |  |
| Low risk pregnancy |  |  | .658 |  | |  | >.99 |  | |  | **<.001** |
| Yes | 842 (56.4) | 652 (43.6) |  | 125 (41.0) | | 180 (59.0) |  | 190 (54.3) | | 160 (45.7) |  |
| No | 684 (57.3) | 510 (42.7) |  | 68 (40.5) | | 100 (59.5) |  | 413 (69.3) | | 183 (30.7) |  |
| **Birth** |  |  |  |  | |  |  |  | |  |  |
| Delivery hour |  |  | .690 |  | |  | .732 |  | |  | .584 |
| Day 7:00 am – 8:59 pm | 976 (57.1) | 733 (42.9) |  | 108 (41.7) | | 151 (58.3) |  | 379 (64.5) | | 209 (35.5) |  |
| Night 9:00 pm – 6:59 am | 550 (56.2) | 428 (43.8) |  | 85 (39.7) | | 129 (60.3) |  | 223 (62.5) | | 134 (37.5) |  |
| *NA* | *0* | *1* |  | *0* | | *0* |  | *0* | | *0* |  |
| Labor induction |  |  | **<.001** |  | |  | .386 |  | |  |  |
| Spontaneous labor | 628 (44.7) | 777 (55.3) |  | 143 (42.2) | | 196 (57.8) |  |  | |  |  |
| Induction with oxytocin | 394 (75.3) | 129 (24.7) |  | 50 (37.3) | | 84 (62.7) |  |  | |  |  |
| Cervical ripening | 502 (66.2) | 256 (33.8) |  |  |  |  |  |  | |  |  |
| *NA* | *2* | *0* |  | *0* | | *0* |  |  | |  |  |
| Cervical dilation at oxytocin initiation (cm) |  |  | **<.001** |  | |  |  |  | |  |  |
| 0-2 | 648 (74.2) | 225 (25.8) |  |  | |  |  |  | |  |  |
| 3-4 | 372 (59.5) | 253 (40.5) |  |  | |  |  |  | |  |  |
| 5-9 | 281 (45.1) | 342 (54.9) |  |  | |  |  |  | |  |  |
| 10 | 192 (38.7) | 304 (61.3) |  |  | |  |  |  | |  |  |
| *NA* | *33* | *38* |  |  | |  |  |  | |  |  |
| Epidural analgesia during labor |  |  |  |  | |  | .210 |  | |  |  |
| Yes |  |  |  | 182 (41.7) | | 254 (58.3) |  |  | |  |  |
| No |  |  |  | 11 (29.7) | | 26 (70.3) |  |  | |  |  |
| Practitioner at birth |  |  |  |  | |  | **<.001** |  | |  |  |
| Spontaneous birth with a midwife |  |  |  | 89 (53.3) | | 78 (46.7) |  |  | |  |  |
| Spontaneous birth with an obstetrician |  |  |  | 15 (31.9) | | 32 (68.1) |  |  | |  |  |
| Instrumental birth with an obstetrician |  |  |  | 88 (34.4) | | 168 (65.6) |  |  | |  |  |
| *NA* |  |  |  | *1* | | *2* |  |  | |  |  |
| Timing of cesarean birth |  |  |  |  | |  |  |  | |  | **.043** |
| Cesarean before labor |  |  |  |  | |  |  | 127 (70.6) | | 53 (29.4) |  |
| Cesarean during labor |  |  |  |  | |  |  | 476 (62.1) | | 290 (37.9) |  |
| Indication for cesarean section |  |  |  |  | |  |  |  | |  | **<.001** |
| Fetal |  |  |  |  | |  |  | 241 (55.4) | | 194 (44.6) |  |
| Obstetric |  |  |  |  | |  |  | 310 (71.8) | | 122 (28.2) |  |
| Maternal |  |  |  |  | |  |  | 40 (63.5) | | 23 (36.5) |  |
| *NA* |  |  |  |  | |  |  | *12* | | *4* |  |
| **Child** |  |  |  |  | |  |  |  | |  |  |
| Gestational age at birth (weeks) |  |  | >.99 |  | |  | .862 |  | |  | .067 |
| <37 | 59 (56.7) | 45 (43.3) |  | 5 (38.5) | | 8 (61.5) |  | 84 (71.8) | | 33 (28.2) |  |
| ≥37 | 1,467 (56.8) | 1,117 (43.2) |  | 188 (40.9) | | 272 (59.1) |  | 519 (62.6) | | 310 (37.4) |  |
| Poor neonatal outcome |  |  |  |  | |  | .911 |  | |  | .077 |
| No |  |  |  | 137 (39.6) | | 209 (60.4) |  | 365 (65.2) | | 195 (34.8) |  |
| Yes |  |  |  | 24 (41.4) | | 34 (58.6) |  | 160 (58.6) | | 113 (41.4) |  |
| *NA* |  |  |  | *32* | | *37* |  | *78* | | *35* |  |
| **Facility** |  |  |  |  | |  |  |  | |  |  |
| Level of care |  |  | .622 |  | |  | **.027** |  | |  | .452 |
| I | 327 (55.4) | 263 (44.6) |  | 36 (33.6) | | 71 (66.4) |  | 130 (60.2) | | 86 (39.8) |  |
| IIa and IIb | 798 (56.7) | 610 (43.3) |  | 95 (39.1) | | 148 (60.9) |  | 305 (64.5) | | 168 (35.5) |  |
| III | 401 (58.1) | 289 (41.9) |  | 62 (50.4) | | 61 (49.6) |  | 168 (65.4) | | 89 (34.6) |  |
| Maternity unit legal status and volume (births/year) |  |  | .301 |  | |  | **.001** |  | |  | .642 |
| Public university hospital | 295 (58.5) | 209 (41.5) |  | 47 (54.0) | | 40 (46.0) |  | 132 (68.0) | | 62 (32.0) |  |
| Other public* ≥2000 | 415 (59.2) | 286 (40.8) |  | 60 (49.6) | | 61 (50.4) |  | 157 (63.6) | | 90 (36.4) |  |
| Other public* <2000 | 467 (54.1) | 396 (45.9) |  | 54 (32.0) | | 115 (68.0) |  | 192 (62.5) | | 115 (37.5) |  |
| Private ≥2000 | 102 (56.4) | 79 (43.6) |  | 7 (33.3) | | 14 (66.7) |  | 32 (65.3) | | 17 (34.7) |  |
| Private <2000 | 247 (56.3) | 192 (43.7) |  | 25 (33.3) | | 50 (66.7) |  | 90 (60.4) | | 59 (39.6) |  |

*BMI: body mass index, CR: consent requested, NA: not available, NCR: no consent requested, SD: standard deviation*

° Not including prophylactic oxytocin dose at delivery of shoulder

* Other public category includes public facilities (except for teaching hospitals) and private nonprofit facilities performing public service.

Chi-square test or Fisher's exact test.
